# Cardiorespiratory and vascular function outcomes following 4 weeks of single-sprint training

**DOI:** 10.3389/fphys.2026.1741749

**Published:** 2026-05-29

**Authors:** Matthew A. Chatlaong, John P. Bentley, Hannah C. Dowell, Orlandria J. Smith, Matthew B. Jessee

**Affiliations:** 1 Exercise Science, School of Human Services, University of Cincinnati, Cincinnati, OH, United States; 2 Applied Human Health and Physical Function Laboratory, Department of Health, Exercise Science, and Recreation Management, University of Mississippi, University, MS, United States; 3 Department of Pharmacy Administration, School of Pharmacy, University of Mississippi, University, MS, United States

**Keywords:** endothelial, hemodynamic, macrovascular, maximal oxygen uptake, microvascular, ventilatory thresholds

## Abstract

**Introduction:**

It is unknown if brief intense exercise bouts are sufficient to improve vascular function.

**Methods:**

Healthy participants (n=46) ages 18–35 were allocated to either a training (n=24) or control (n=22) group. Training consisted of a 20s maximal cycling sprint (no warm-up or cool-down) 3 days/week, while control received no training intervention. Testing occurred at pre-, mid- (2 weeks), and post-intervention (4 weeks). Post occlusive reactive hyperemia (PORH), brachial flow-mediated dilation, and passive leg movement hyperemia were used to assess vascular function. Graded exercise testing was used to test cardiorespiratory fitness. A Bayesian repeated measures ANOVA approach with effect estimation was used. Results are mean change (SD) from pre-to-post testing sessions with BF_incl_ in reference to group x time interaction.

**Results:**

There was no evidence of a training effect for vascular and cardiorespiratory outcomes. Changes in forearm PORH via ultrasound (cm/s) were 0.35 (11.61) for training and -1.59 (10.06) for control (BF_incl_ = 0.177) and via near-infrared spectroscopy (StO_2_%/s) were -0.03 (0.61) for training and -0.13 (0.49) for control (BF_incl_ = 0.161). Changes in leg PORH via ultrasound (cm/s) were -1.20 (10.88) for training and 3.51 (15.92) for control (BF_incl_ = 1.070) and via near-infrared spectroscopy (StO_2_%/s) were 0.15 (0.38) for training and -0.03 (0.29) for control (BF_incl_ = 0.492). Changes in maximal oxygen uptake (L/min) were 0.00 (0.19) for training and 0.05 (0.14) for control (BF_incl_ = 0.394).

**Conclusion:**

Vascular and cardiorespiratory function did not show meaningful improvement, establishing an important basis for future investigation of vascular adaptations to brief intense exercise bouts.

## Introduction

The progression of early vascular aging promotes the development of hypertension and elevated cardiovascular disease (CVD) risk with age (Kaess et al., 2012; Olsen et al., 2016). Vascular endothelial dysfunction has been established as a mediator of macro- (large artery) and micro- (small vessel) vascular dysfunction (Kaess et al., 2012) (for reviews, see Matsuzawa and Lerman (2014); Olsen et al. (2016); Vanhoutte et al. (2017)), compromising circulatory control and anti-atherogenic protection of the adjacent artery wall (for review, see Förstermann et al. (2017) and Gimbrone and García-Cardeña (2016)). In young adults (age 24–39 years), there is evidence that childhood exposure to traditional CVD risk factors is associated with adult carotid intima-media thickness, though only among individuals with impaired but not preserved endothelial function (assessed by brachial artery flow-mediated dilation (FMD)) (Juonala et al., 2004). Other longitudinal studies indicate that endothelial dysfunction precedes and predicts future development of hypertension (Kaess et al., 2012) and cardiovascular events (Shechter et al., 2014). Indeed, these findings align with the idea that gradual development of macro- and micro-vascular dysfunction, result in subclinical vessel and tissue damage that eventually manifests in clinical presentations of CVD (for reviews, see Nilsson et al. (2009); Olsen et al. (2016)).

Endothelial dysfunction is characterized by reduced nitric oxide (NO) bioavailability which may limit the capacity of the endothelium to evoke vasodilation (Taddei et al., 1997, 1998; Vanhoutte et al., 2017). NO-mediated vasodilation helps regulate resistance vessel tone and blood pressure through interacting with vasoconstrictor input (e.g., sympathetic nerve input) (Joyner et al., 2010). This is exemplified by dose-dependent increases in resting mean arterial pressure with pharmacological inhibition of NO synthase being greater in individuals with high (~15 mmHg) versus low (~9 mmHg) resting muscle sympathetic nerve activity (Charkoudian et al., 2006). With regard to developing hypertension, this could suggest certain individuals have heightened sensitivity to disrupted NO-mediated vasodilation (Charkoudian et al., 2006; Joyner et al., 2010).

Exercise training is recognized as a potent stimulus for vascular remodeling (Green et al., 2017), and improved endothelial function may partially mediate cardiovascular risk reduction from training interventions (Green et al., 2008; Joyner and Green, 2009). Overall, meta-analytic findings indicate that several endurance and resistance training modalities appear to have positive effects on vascular endothelial function assessed via brachial FMD (Ashor et al., 2015; Silva et al., 2021; Khalafi et al., 2022; Shivgulam et al., 2023). Reported effects appear typically between 2 to 4 unit increases in %FMD across a wide range of participant and intervention characteristics, and some analyses conclude that greater intensity of endurance exercise modalities may be beneficial in certain populations (Ashor et al., 2015; Khalafi et al., 2022). Exercise-induced increases in blood flow amplify mechanical forces on vessel walls, i.e., providing “hemodynamic stimuli” for acute vascular responses and long-term remodeling (for review, see Green et al. (2017)). Specifically, increased luminal shear stress during exercise causes endothelial NO release to support normal circulatory responses (Green et al., 2002, 2017). Recurring systemic endothelial NO release throughout a training intervention may enhance the systemic capacity for endothelial NO production and NO bioavailability (Green et al., 2002). For example, it has been shown that 4 weeks of exercise training led to a doubling of endothelial nitric oxide synthase (eNOS) protein content in the left internal mammary artery of patients with coronary artery disease (Hambrecht et al., 2003), and that 6-weeks of training resulted in ~14-36% increases in eNOS protein content of micro vessels from the vastus lateralis in young sedentary males (Cocks et al., 2013). The role of exercise-induced shear stress in stimulating endothelial adaptations is further evidenced by studies using pneumatic cuffing on the forearm to unilaterally attenuate brachial artery shear stress throughout exercise training interventions (Tinken et al., 2010; Birk et al., 2012). These studies have demonstrated that training-induced improvements in brachial FMD may be blunted in the cuffed arm. Collectively, exercise-induced shear stress appears to drive favorable vascular adaptations.

While positive effects of many exercise modalities on vascular endothelial function have been established, investigations focused on sprint interval training in young adults are limited to a handful of small studies with some (Rakobowchuk et al., 2008; Petrick et al., 2021), but not all (Shenouda et al., 2017), indicating improvements in FMD outcomes and others demonstrating increased microvascular eNOS content and capillarization in the vastus lateralis (Cocks et al., 2013, 2016). Despite growing interest into the health benefits of these modalities, especially those designed to incorporate brief intense exercise efforts in lieu of entire workout sessions (Islam et al., 2022; Weston et al., 2025), the impact on endothelial function remains unclear. Many investigations have primarily focused on cardioprotective benefits mediated by improved maximal oxygen uptake (VO_2max_). For example, an intriguing line of studies investigating the dose-response relationship of health benefits from brief sprint exercise bouts has attempted to establish a minimal threshold of exercise duration that can elicit improved VO_2max_ in healthy, sedentary adults (Metcalfe et al., 2012, 2016; Songsorn et al., 2016; Nalçakan et al., 2017; Vollaard and Metcalfe, 2017). In attempting to maximize time-efficiency and lessen the strenuous nature of repeated sprint efforts, these studies have demonstrated that training interventions utilizing 2 x 20-second sprint efforts in 10-minute bouts, three times per week, consistently improve VO_2max_ (e.g., typically by ~7-12%) (Metcalfe et al., 2012, 2016; Cuddy et al., 2019; Metcalfe et al., 2020). However, reducing the volume of each training session to a single isolated 20-second sprint effort (i.e., removing warm-up, active recovery between sprints, and cool-down) resulted in a lack of improvement in VO_2max_ (Songsorn et al., 2016). Interestingly, it was reported that training with the single isolated sprint effort elicited an average of ~90% maximum heart rate (Songsorn et al., 2016), suggesting that a marked systemic hemodynamic shear stress stimulus could result, and thus we considered whether this type of exercise could improve endothelial function. Observations from our laboratory during an acute exercise bout indicate that with 20-second sprint efforts performed without warm-up, active recovery between sprints, or cool-down, robust (but transient) increases in brachial artery shear rate occurred both the initial and second sprint (e.g., peak shear rate values ~ 175^-s^) (Chatlaong et al., 2025). To our knowledge, it remains unclear if the type of shear stimulus observed acutely following a single sprint effort could drive vascular adaptations following a training intervention (Chatlaong et al., 2025). If so, training consisting of a single isolated sprint effort could present a novel brief exercise programming approach with cardioprotective benefits. Future research could then investigate the applicability in wider contexts (e.g., with different exercise modes, settings, and populations) alongside other forms of isolated intense exercise.

Accordingly, a considerable knowledge gap remains around whether vascular function could improve independently of Vo_2max_ with the single-sprint training modality, and there does not appear to be further study of this modality after being established as an insufficient stimulus for improving VO_2max_ (Songsorn et al., 2016, 2017). Thus, the main objectives of this study were: 1) to assess potential effects of the single-sprint training modality on local and/or systemic vascular function in young, sedentary individuals and 2) to further investigate potential effects on maximal oxygen uptake and components of oxygen transport. We hypothesized that evidence would favor improvements in vascular function outcomes, ventilatory thresholds, and thigh muscle oxidative capacity, but not whole-body cardiorespiratory fitness following the single-sprint training intervention.

## Materials and methods

### Participants

Fifty participants were enrolled, however, 4 withdrew due to scheduling conflicts (3 before pre-intervention testing, and 1, from the sprint group, before mid-intervention testing). Consequently 46 young, healthy participants (female and male both n = 23) completed the study (**Table 1**). To be included, participants had to be: 1) age 18–35 years, 2) free from injury or illness affecting any testing procedures or training, 3) screened via the PAR-Q + (Warburton et al., 2011), 4) not taking medications affecting heart rate or blood pressure, 5) not regular nicotine users in the prior 6 months. In the prior 6 months, participants had not engaged in any planned or routine endurance or resistance exercise training for at least a 2-week window and did not accumulate > 150 min of moderate intensity physical activity per week through activities such as walking, jogging, or work (self-reported). Participants agreed to maintain consistent dietary intake, sleep behaviors, and physical activity levels aside from the study.

**Table 1:** Participant characteristics by group and sex.

Characteristic	Control		Training	
	Female n = 11	Male n = 11	Female n = 12	Male n = 12
Age (years)	22.5 (4.4)	28.1 (4.1)	21.6 (4.5)	26.3 (4.8)
Height (cm)	163.8 (5.2)	175.0 (10.2)	161.6 (8.2)	173.4 (7.6)
Mass (kg)	63.5 (7.8)	79.5 (14.1)	63.9 (15.4)	77.5 (25.0)

Values are mean (SD). Characteristics were not statistically compared between strata.

This study was developed as part of a pilot projects program, pre-registered on clinicaltrials.gov (NCT05727332). Frequentist null hypothesis significance testing was not used and thus no *a priori* power analysis was conducted. Sample size was based on: 1) using a Bayesian statistical approach to inform future work, 2) similar sprint interval training studies where across 38 studies the average sample size per group was approximately n = 11 (Vollaard et al., 2017), and 3) available resources (Lakens, 2022). As a pilot study, Bayesian estimates can be used for planning larger confirmatory trials or informing analyses in similar studies (Kruschke, 2013; 2015; Chen and Fraser, 2017; Kruschke and Liddell, 2018; van de Schoot et al., 2021). The goal at study onset was to retain a total of 40 participants for these estimates. Based on a prior exercise training study completed at the university (Jessee et al., 2018) a ~13% attrition rate was anticipated (n = 6). This study was approved by the Institutional Review Board at the University of Mississippi (protocol 22-037), and participants gave written consent to take part in the study after being informed of all procedures, risks, and study objectives.

### Study design

**Figure 1:**
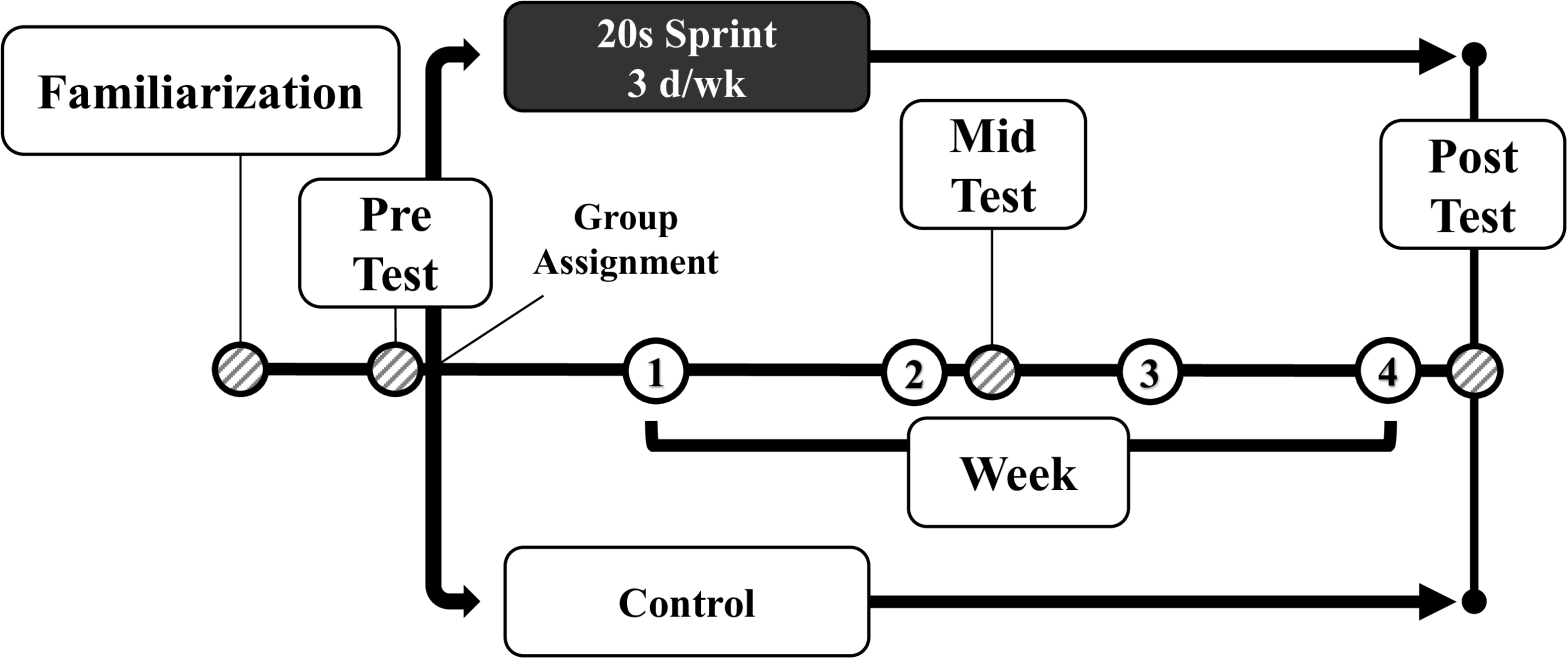
Overview of experimental design.

A 4-week parallel randomized-controlled trial design with a single-sprint training intervention group and a time-matched negative control group was used (Songsorn et al., 2016). The timeline was used to replicate Songsorn et al. (2016), although mid-intervention testing was added to evaluate the time course of potential vascular changes. All participants began the study with a familiarization session, then after at least 48 hours, outcomes were assessed at a pre-intervention testing visit (week 0), then again at mid- (end of week 2) and post-intervention (end of week 4) time points (**Figure 1**). Participants (stratified by sex) were allocated to either the training or control group on a rolling basis after the pre-intervention visit by using a random draw procedure assigning n = 24 to the intervention n = 22 to the control group.

#### Training intervention

Training bouts consisted of a 20-second sprint effort with no warm-up or cool-down, performed separately 3 days per week (Songsorn et al., 2016). Per the ergometer (Excalibur Sport, Lode) manufacturer’s directions for sprint testing, a resistance level of 0.67 NM/kg of body weight for females and 0.70 NM/kg for males was applied. Each sprint began with the resistance load on the ergometer, and training intervention group participants were instructed to 1) pedal as powerfully as possible to overcome the resistance as quickly as possible, 2) get their feet turning as fast as possible, and 3) continue pedaling as hard as possible preventing cadence from slowing throughout the 20 seconds. Each session, participants were encouraged to beat their previous peak values, and strong verbal encouragement was given throughout.

Each week, resistance was progressed by a torque factor of 0.02 NM/kg of initial body weight. Training sessions were separated by 1–2 days each week unless scheduling on consecutive days was necessary. Only 2 consecutive days were scheduled within a week. If needed, a third training session could made up the following week, granted it did not necessitate more than 2 consecutive training days.

Pre- and post-intervention visits were conducted within 48–72 hours of the first and last days of the 4-week intervention period, and the mid-intervention visit was at the end of week 2, within 48–72 hours of the last exercise session. The 48–72 hour time frame between exercise bouts and testing visits was to avoid capturing acute effects of exercise on vascular outcomes (Limberg et al., 2019), as chronic adaptations were the intended endpoint. We reasoned that a 72-hour upper limit would avoid disrupting study timeline and/or the onset of detraining (Boyle et al., 2013). For each participant, testing visits were matched for time of day (within +/- 2 hours of their initial pre-intervention testing time) (Limberg et al., 2019; Thijssen et al., 2019). No attempt was made to account for menstrual cycle phase as rolling enrollment and random group allocation should have helped to avoid systematic effects. Nonetheless, a calendar counting based phase identification was conducted at study onset for reporting purposes (Janse De Jonge et al., 2019). The distribution of participants beginning the study in each phase, having irregular menstrual cycles (i.e., not 28–32 d), and using hormonal birth control was determined. Follicular phase was defined by days 1–14, and luteal phase as days 15-28, where day 1 was determined by the last onset of menstruation (Janse De Jonge et al., 2019).

In this study, several non-invasive measures were used to assess distinct aspects of vascular and cardiometabolic function. At testing visits, the following outcomes were assessed (in order): body height and mass, resting blood pressure, forearm post-occlusive reactive hyperemia (PORH) and brachial artery FMD, passive leg movement hyperemia (PLM), thigh PORH, non-invasive muscle oxidative capacity of the quadriceps, and cardiorespiratory fitness via a graded exercise test. Graded exercise testing was only completed at pre- and post-intervention visits to avoid an additional maximal exercise session during the intervention. For testing, participants were instructed to refrain from the following: incidental alcohol and nicotine use for 24 hours, elevation in physical activity for 24 hours, caffeine use for at least 8 hours, taking new vitamins or supplements within 72 hours, and food and drinks aside from water for 4 hours (Limberg et al., 2019).

### Study outcomes

#### Body height and mass

Height was measured via stadiometer (213, Seca) and mass was assessed at pre- and post-intervention testing visits using a digital scale (498KL, Health O Meter).

#### Blood pressure

Resting blood pressure was assessed in the supine position after a 10-minute rest period via an automated cuff system (BP742N, OMRON). To ensure stable values, multiple measures were taken, each separated by one minute, until systolic and diastolic blood pressure of two measurements differed by 5 mmHg or less. Those two measurements were then averaged and used for analyses.

#### Forearm post-occlusive reactive hyperemia

While remaining supine, forearm PORH and brachial artery FMD (described later) were assessed simultaneously following a 5-minute arterial occlusion via a pneumatic cuff (SC5D, Hokanson) on the right forearm. Although assessed simultaneously, PORH evaluates flow and velocity related outcomes driven by microvascular responsiveness in small forearm vessels (Limberg et al., 2019; Rosenberry and Nelson, 2020) and FMD measures conduit artery dilation mediated by NO dependent endothelial function in the brachial artery (Thijssen et al., 2019). PORH was assessed using both ultrasound and near-infrared spectroscopy (NIRS) methods (Rosenberry and Nelson, 2020) as has been done previously in our laboratory (Chatlaong et al., 2024). Duplex ultrasound (HS60, Samsung) scans were made using a gel-coated linear array ultrasound probe (LA3-14, Samsung) to image the brachial artery approximately 2–3 cm above the antecubital space with care taken to avoid artery bifurcations. To quantify PORH which occurs rapidly after cuff release, peak blood velocity was determined (Rosenberry and Nelson, 2020) from a 30-second video recording analyzed offline with validated FloWave.US software (Coolbaugh et al., 2016). A 3-second moving average was applied before determining the highest time-average mean velocity value (Chatlaong et al., 2024). Hyperemic blood velocity was chosen as the outcome of interest, as it is considered a valid indicator of cardiovascular disease risk as demonstrated in several large cohort studies (Mitchell et al., 2005; Philpott et al., 2009; Anderson et al., 2011; Cooper et al., 2016, 2020). Interestingly, some evidence suggests hyperemic blood velocity may provide additional predictive value for CVD risk beyond FMD (Philpott et al., 2009), and it is suggested that peak velocity is proportional to the capacity of distal resistance vasculature to dilate (Cooper et al., 2020). For all Doppler ultrasound blood velocity measures in this study, guidelines were closely followed (Limberg et al., 2019). Specifically, sample volume encompassed the entire lumen, beam insonation angle was less than 60 degrees, 2D gain, pulse wave gain, sweep speed, and sample volume settings were reproduced in subsequent visits (Limberg et al., 2019). In previous studies, we have demonstrated strong test-retest correlations for Doppler ultrasound measures at rest and following exercise (Stanford et al., 2022; Chatlaong et al., 2025). The same experienced investigator performed all ultrasound scans and offline analyses in this study, and video recordings from the pre-intervention visit were referenced to compare tissue landmarks in subsequent visits.

The NIRS method utilized a portable device (PortaMon MKII, Artinis) to record oxygen saturation of the forearm flexor muscles at the midpoint between the medial epicondyle of the humerus and the radial styloid process. Linear regression was used to determine the slope of the initial linear rise in tissue oxygen saturation (StO_2_) over ~10 seconds following cuff release (McLay et al., 2016a; Barstow, 2019; Rosenberry and Nelson, 2020). The fitting window was adjusted as necessary to ensure that only the initial linear rise was fitted with no influence of movement artifact from rapid cuff release (Barstow, 2019). Adipose tissue thickness (ATT) was assessed at the measurement site via B mode ultrasound. Based on estimating NIRS signal depth as one-half the distance between the light source and detector (Barstow, 2019), adipose tissue thickness of > 2 cm would indicate that underlying skeletal muscle was too deep for measurement with the PortaMon. For the occlusion period, 130% of the arterial occlusion pressure (AOP) was applied in the cuff as previously described (Chatlaong et al., 2024). NIRS-derived PORH is considered a valid technique for the assessment of microvascular reactivity (McLay et al., 2016a; Barstow, 2019), typically demonstrating good test-retest reliability (McLay et al., 2016b; Iannetta et al., 2019; McGranahan et al., 2023) though not in all studies (Rogers et al., 2023).

#### Brachial flow-mediated dilation

At baseline, B mode ultrasound was used for a 1-minute high-quality scan of brachial artery diameter, then the cuff was inflated (5-min), then rapidly deflated (Thijssen et al., 2019). B mode scanning was interrupted for 30 seconds to capture blood velocity upon cuff release (see Forearm PORH), then resumed to record the artery’s dilatory response until five minutes post-deflation. This timing was based on peak FMD being observed about 1–3 minutes post-deflation in previous studies (Black et al., 2008; Thijssen et al., 2008). Video recordings were analyzed offline using automated edge detection and wall tracking software (FloWave.US). Baseline artery diameter was quantified as the average throughout the 1-minute before cuff inflation. FMD was determined by the peak change in artery diameter from baseline after applying a 3-second moving average. Relative FMD (% change) was also calculated. FMD is considered a valid assessment of conduit artery NO mediated endothelial function (Green et al., 2014), being predictive of cardiovascular disease risk in large cohort studies (Inaba et al., 2010; Kaess et al., 2012; Shechter et al., 2014). Several studies (Ghiadoni et al., 2012; Charakida et al., 2013; Marôco et al., 2022; Daniele et al., 2024), though not all (Shenouda et al., 2015; McLay et al., 2016b), demonstrate good test-retest reliability for FMD.

#### Passive leg movement hyperemia

Endothelial NO mediated micro vessel function was assessed in the leg via passive leg movement hyperemia (PLM) (Mortensen et al., 2012; Gifford and Richardson, 2017; Limberg et al., 2019). Based on current guidelines, the participant was tested sitting upright, maintaining complete relaxation of trunk and leg muscles while a dynamometer (System 3, Biodex) was used to passively flex and extend the knee through a 90-degree range of motion at a controlled rate of 1hz for 1 minute (Gifford and Richardson, 2017). Participants practiced the protocol thoroughly during the familiarization visit, and at each subsequent visit, emphasis was placed on complete relaxation throughout. The increase in leg blood flow was assessed via duplex ultrasound in the common femoral artery at baseline and during PLM (Gifford and Richardson, 2017). Baseline blood velocity was measured for 2 minutes, then a 1-minute high-quality B mode video recording was used for artery diameter. Care was taken to ensure that the artery was imaged 2–3 cm proximal to the bifurcation (Gifford and Richardson, 2017). Baseline diameter was assumed to remain constant throughout the trial (Gifford and Richardson, 2017). The highest 3-second averaged blood flow value occurring throughout PLM was used to calculate a change score from the average baseline flow (Gifford and Richardson, 2017). PLM is considered a valid assessment of microvascular reactivity, dependent on endothelial NO release (Mortensen et al., 2012; Groot et al., 2015; Gifford and Richardson, 2017; Limberg et al., 2019) with studies demonstrating good test retest reliability (Lew et al., 2021; Groot et al., 2022).

#### Thigh post-occlusive reactive hyperemia

Similar methodology to forearm PORH was used in the thigh. A 12 cm pneumatic cuff (SC12D, Hokanson) was fitted to the upper thigh of the right leg, and the NIRS device was placed over the vastus lateralis 2/3 the distance between the anterior superior iliac spine and the lateral border of the patella. AOP was determined as the cuff pressure occluding blood flow at the posterior tibial artery near the ankle (assessed via handheld Doppler). A 5-minute occlusion at 130% AOP was used to induce PORH while blood velocity was recorded in the common femoral artery. Similarly to the arm, PORH was determined via the highest 3-second velocity value and StO_2_ slope after cuff release.

#### Non-invasive muscle oxidative capacity

**Figure 2:**
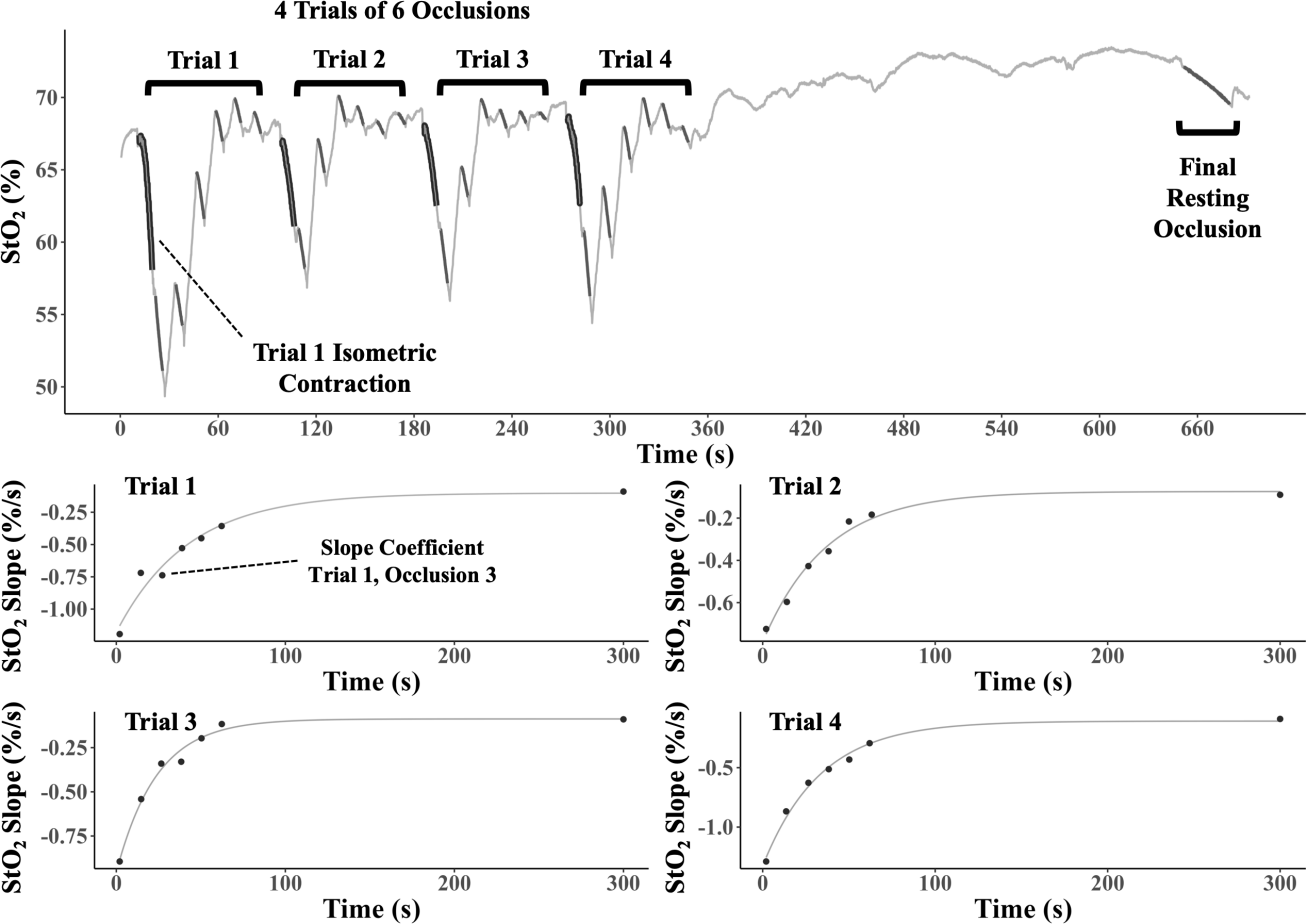
Example data for non-invasive muscle oxidative capacity protocol. Top panel shows StO_2,_ tissue oxygen saturation signal throughout the 4 trials of muscle contractions followed by 6 x 5s on/5s off cuff inflations. The four bottom panels show the monoexponential recovery of slope coefficients calculated from each occlusion. The final data point in each of the curves is the slope from the “Final Resting Occlusion” shown in the top panel.


Muscle oxidative capacity was assessed in the vastus lateralis of the left leg using the protocol from Sumner et al. (2020) (**Figure 2**). The right leg was not assessed given potential carry over effects from previous PORH testing. The protocol involved 4 trials of a 10-second submaximal (self-estimated 30-50% of maximum effort) isometric knee extension (knee in 90 degrees of flexion) to increase metabolic rate (Ryan et al., 2013), followed by a series of 6 brief (5s inflated/5s deflated at 130% AOP) occlusions (**Figure 2**). After the 4^th^ trial of occlusions, the participant rested passively for 5 minutes followed by a final 30-second occlusion (**Figure 2**) (Sumner et al., 2020). During each occlusion, the slope of the StO_2_% signal was used as a marker of muscle oxygen consumption. The rate of change in slope coefficients during each trial was fitted via a mono-exponential curve (Motobe et al., 2004; Sumner et al., 2020), using non-linear least squares regression in R software (2021) and the following equation (Sumner et al., 2020):

$$y(t)=End-\;\Delta \times e^{-k\cdot t}$$

where *y(t)* represents metabolic rate at a given time, *End* is the estimated end-exercise metabolic rate, Δ is the estimated change in metabolic rate between *End* and full recovery, *k* is the fitting rate constant, and *t* is time. The time constant (1/k), reported in seconds, was used for analysis. Curves were fitted individually for each trial and averaged. The final resting occlusion taken 5 minutes after trial 4 was used in fitting each curve (Sumner et al., 2020). NIRS-derived muscle oxidative capacity measures have been shown to be repeatable (Beever et al., 2020; Fennell et al., 2023) and are considered a valid assessment of oxidative capacity (Ryan et al., 2014; Pilotto et al., 2022; Tripp et al., 2023).

#### Graded exercise testing

Maximal oxygen uptake and ventilatory thresholds were assed via graded exercise testing on an electronically braked cycle ergometer (Excalibur Sport, Lode) in accordance with current recommendations (Balady et al., 2010; ACSM, 2022). The protocol was performed in full during the familiarization visit. Expired gas was collected (True One 2400, Parvomedics) and heart rate was monitored (H10, Polar) throughout. After a 5-minute warm-up at 50W and a short rest period, participants completed a ramp-incremental protocol beginning at 50W increasing by 15 W/min for females and 25 W/min for males until volitional exhaustion. Ramp rates were selected to elicit a test length of 6–12 minutes at volitional exhaustion and a smooth increase in VO_2_ for ventilatory threshold detection (Balady et al., 2010; Wasserman et al., 2011). Volitional exhaustion was determined by: 1) the participant stopping, or 2) the participant being unable to maintain at least 50 revolutions per minute. Participants rated their perceived exertion each minute using the 6–20 Borg RPE scale (Balady et al., 2010).

Data were 20-second bin averaged (Balady et al., 2010), then VO_2max_ was defined as the highest value. VO_2max_ was considered achieved if 2 or more of the following criteria were met: 1) RPE ≥ 18 (Balady et al., 2010), 2) ≤ 150 mL/min increase in VO_2_ with increasing workload (Wasserman et al., 2011), 3) peak respiratory exchange ratio (RER) ≥ 1.10 (Balady et al., 2010; Kaminsky et al., 2017), 4) peak heart rate ≤ 10 beats per minute from age predicted max (Gellish et al., 2007). The time to exhaustion from the test was used to quantify exercise tolerance. Ventilatory thresholds delineating metabolic domains (Black et al., 2017; Burnley and Jones, 2018) were determined via a piecewise regression approach using Winbreak software (Version 3.7) (Ekkekakis et al., 2008) with visual confirmation. The VO_2_ at the respiratory compensation point (RCP) was estimated via a break point in the VE - VCO_2_ relationship, then the ventilation threshold (VT) was estimated via a break point in the minute ventilation (VE) - VO_2_ relationship occurring below the RCP. These signals were used based on the visual ventilatory equivalents method (Balady et al., 2010; Wasserman et al., 2011). The ventilatory equivalents method is considered a valid and reliable technique to assess ventilatory thresholds (Amann et al., 2004).

### Statistical analyses

#### Primary analyses

Analyses were based on: 1) Bayesian parameter estimation for incorporation in future studies and 2) Bayes factors comparing evidence in favor of, or against, treatment effects. For parameter/treatment effect estimation, Bayesian linear mixed models with factors of group and time were fitted using the rstanarm package for R software with default prior distributions (Goodrich et al., 2020). Baseline values were used as covariates to adjust for random differences (Senn, 2006). Posterior distributions of model parameters were sampled via the Markov chain Monte Carlo (MCMC) method using 4 parallel Markov chains, with 50,000 iterations per chain, 2,000 of which were dedicated to warmup (McElreath, 2020). Posterior distributions of treatment effect parameters were described by their median (serving as a point estimate), standard deviation, and 95% highest density credible interval (CI) using the describe_posterior function in the bayestestR package (Makowski et al., 2019). Standardized parameter estimates (Cohen’s *d*) were calculated using model-based SD for each outcome (Nalborczyk et al., 2019) and are presented in **Supplementary Materials** (**Supplementary Table S1**).

Bayes factors were obtained via a Bayesian repeated measures ANOVA model comparison approach (Rouder et al., 2016; van den Bergh et al., 2020) implemented in JASP software (version 0.19) using default priors (Rouder et al., 2012). Models were fitted with factors of group and time with baseline values as covariates (added to the null model). Bayes factors were used to compare the relative probability of candidate models: a null model, those with one or more main effects, or the full factorial model containing the group x time interaction terms (treatment effects). Model comparisons are presented with BF_10_ made relative to the null model, where BF_10_ = 1/3 suggests the null model is 3 times more likely than the given candidate model, and BF_10_ = 3 suggests the candidate model is 3 times more likely than the null (Rouder et al., 2016). Additionally, since the group x time interaction is the focal point (treatment effects), inclusion Bayes factors (BF_incl_) describing the improvement (or penalty) to model probability from including the interaction term(s) are reported (Hinne et al., 2020).^1^ Bayes factors are reported with % error, where lower than 20% is considered acceptable, i.e., highly unlikely to affect conclusions (van Doorn et al., 2021). Interpretation and the strength of evidence denoted by Bayes factors for model comparisons was based on the classification scheme outlined by van Doorn et al. (2021).

#### Subgroup analysis

Treatment effect modification by sex was explored (NIH Policy on Sex as a Biological Variable, 2024). A factor of sex was added to the linear mixed models and ANOVA approach outlined above. For model comparisons, the higher order interaction creates a large model space with combinations of the lower order terms, and thus only BF_incl_ was used to assess whether the group x sex x time interaction terms added to model probability instead of all possible model comparisons (Hinne et al., 2020).^2^ Default priors were again used in all models. Posterior estimates of treatment effects (adjusted for baseline values) are given as summary statistics describing the posterior distribution of linear mixed model parameter estimates. Summaries are denoted by the distribution’s median (point estimate) alongside a 95% highest density credible interval. Given the focus on training-induced changes in the outcomes being the effect of interest (Dankel, 2020), all observed values in results are presented as each group’s change between time points Δ: mean (SD). Observed mean (SD) for each outcome over time are presented in **Supplementary Materials** (**Supplementary Tables S2**-**S4**).

## Results

### Participant characteristics

Descriptive statistics at study onset are shown in **Table 1**. Estimates of menstrual cycle phase and status at study onset are shown in **Table 2**.

**Table 2:** Menstrual cycle status at study onset.

Status	Control n = 11	Training n = 12
Taking Hormonal Birth Control	4	5
Follicular Phase	6	4
Luteal Phase	1	2
Irregular Cycle	0	1

Values are counts of participants. Counts were not statistically compared.

### Training intervention

**Figure 3:**
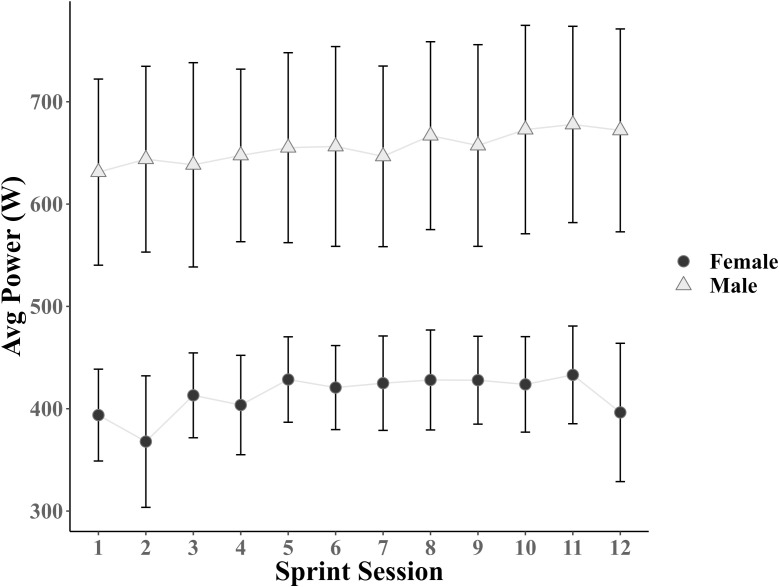
Average power output (W) over the 20-second sprint across all training visits. Circles denote mean values for females and triangles for males. Error bars denote standard deviation. Because no sprints were completed in the control group, no statistical comparisons were made.

All participants in the training intervention group completed all training sessions, and no adverse events were observed or reported by participants. Mean power output across all sessions was 413.5 (19.5) W for females and 655.5 (14.7) W for males. Mean power output for each session is shown in **Figure 3**.

### Forearm post-occlusive reactive hyperemia

**Figure 4:**
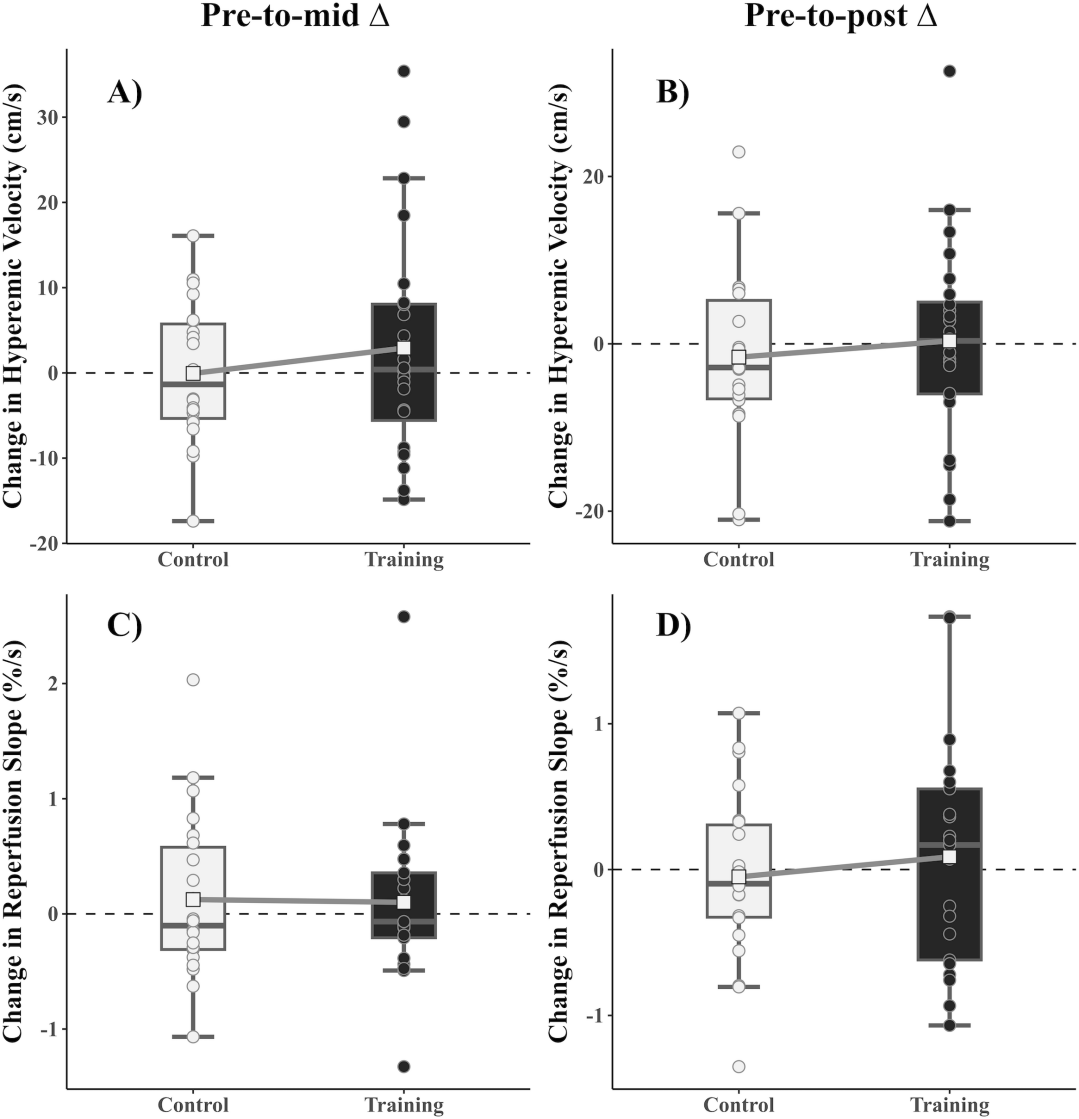
**(A, B)** Changes in brachial peak hyperemic velocity via doppler ultrasound from pre-to-mid **(A)** and pre-to-post **(B)** time points. **(C, D)** Changes in forearm tissue reperfusion slope via NIRS from pre-to-mid **(C)** and pre-to-post time points **(D)**. Dots are individual scores, white squares are group means connected by a line showing mean difference (treatment effect), box limits = 25^th^ and 75^th^ percentiles, center lines = medians, and whiskers = most extreme score within 1.5x the interquartile range.

For both Doppler and NIRS methods (**Figure 4**, **Supplementary Table S2**), there was evidence against a group x time interaction (Doppler: BF_10_ = 0.007, 2.0% error, BF_incl_ = 0.177; NIRS: BF_10_ = 0.005, 3.3% error, BF_incl_ = 0.161). For both methods, estimated treatment effects (**Table 3**) for both pre-to-mid and pre-to-post changes showed considerable credibility around null values. There was evidence against sex-based effect modification for both Doppler (BF_incl_ = 0.185, 2.0% error) and NIRS (BF_incl_ = 0.165, 1.4% error) methods (**Table 3**). For the NIRS method, 2 male participants from the training group were removed from analysis due to poor signal quality on at least one measurement occasion. Forearm ATT was 0.61 (0.22) cm, Min: 0.31, Max: 1.22 cm, less than 2.0 cm for all participants.

**Table 3:** Posterior estimates of treatment effects and modification by sex for systemic/non-local vascular outcomes.

Outcome	Pre-to-mid (Training Δ -Control Δ)	Pre-to-mid modification by sex (M-F)	Pre-to-post (Training Δ -Control Δ)	Pre-to-post modification by sex (M-F)
Brachial Peak Hyperemic Velocity (cm/s)	2.97 [-3.55 to 9.67]	3.14 [-10.29 to 16.59]	1.94 [-4.78 to 8.48]	-0.21 [-13.60 to 13.23]
Forearm NIRS Reperfusion Slope (StO_2_ %/s)	0.00 [-0.37 to 0.35]	-0.25 [-0.97 to 0.47]	0.10 [-0.25 to 0.46]	-0.26 [-0.99 to 0.46]
Brachial FMD (mm)	0.021 [-0.049 to 0.089]	-0.078 [-0.208 to 0.053]	-0.032 [-0.102 to 0.037]	0.061 [-0.071 to 0.190]
Systolic Blood Pressure (mmHg)	0.1 [-4.5 to 4.3]	3.3 [-5.6 to 12.2]	-1.8 [-6.3 to 2.5]	6.8 [-2.1 to 15.6]
Diastolic Blood Pressure (mmHg)	-0.3 [-3.9 to 3.3]	-3.2 [-10.4 to 3.8]	-1.7 [-5.3 to 1.9]	1.7 [-5.5 to 8.7]

Treatment effects are estimates of the training group Δ minus the control group Δ between the denoted time points. Modification by sex reflects the difference in the treatment effect between males and females for the denoted time point (i.e., training group Δ – control group Δ for males minus the training group Δ – control group Δ for females). Values are the median of the posterior effect distribution [95% credible interval]. Note that for some variables a reduction and negative value for treatment effect would be considered favorable (e.g., blood pressure). FMD, flow-mediated dilation; NIRS, near-infrared spectroscopy; StO_2,_ tissue oxygen saturation.

### Brachial FMD

There was evidence against a group x time interaction (BF_10_ = 0.014, 10.8% error, BF_incl_ = 0.147), and inconclusive findings for effect modification by sex (BF_incl_ = 1.322, 18.8% error). Estimated treatment effects are shown in **Table 3**. Observed changes in absolute (mm) and relative (%) FMD from pre-to-mid were control Δ: -0.035 (0.142) mm and -1.06 (3.75) %; training Δ: -0.014 (0.096) mm and -0.33 (2.70) %, and from pre-to-post control Δ: -0.010 (0.108) mm and -0.46 (3.22) %; training Δ: -0.042 (0.121) mm and -1.24 (3.350) % (**Supplementary Table S2**).

### Resting blood pressure

For both systolic (SBP) and diastolic (DBP) blood pressure, there was evidence against a group x time interaction (SBP: BF_10_ = 0.005, 1.1% error, BF_incl_ = 0.170, DBP: BF_10_ = 0.011, 0.6% error, BF_incl_ = 0.181), and evidence favored no effect modification by sex (SBP: BF_incl_ = 0.464, 1.3% error, DBP: BF_incl_ = 0.418, 3.0% error). Estimated treatment effects are shown in **Table 3**. Observed changes in SBP from pre-to-mid were control Δ: -1.1 (9.7) mmHg; training Δ: -1.2 (6.8) mmHg and DBP were control Δ: -1.2 (7.2) mmHg; training Δ: -1.5 (6.2) mmHg. For pre-to-post, observed changes in SBP were control Δ: 0.4 (8.1) mmHg; training Δ: -1.4 (7.3) mmHg and DBP were control Δ: -0.2 (5.6) mmHg; training Δ: -1.9 (6.3) mmHg (**Supplementary Table S2**).

### Thigh post-occlusive reactive hyperemia

**Figure 5:**
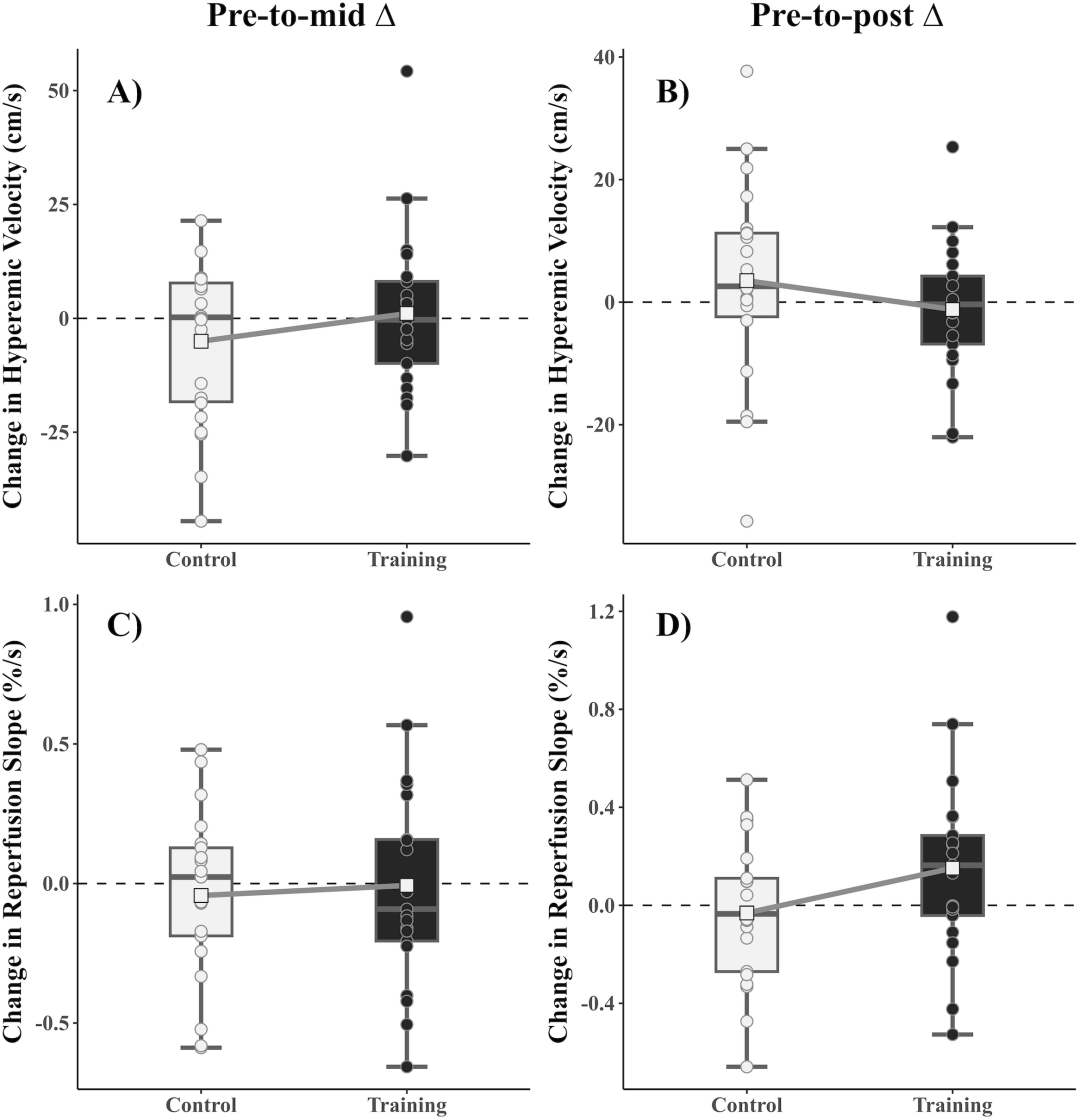
**(A, B)** Changes in femoral peak hyperemic velocity via doppler ultrasound from pre-to-mid **(A)** and pre-to-post **(B)** time points. **(C, D)** Changes in thigh tissue reperfusion slope via NIRS from pre-to-mid **(C)** and pre-to-post **(D)** time points. Dots are individual scores, white squares are group means connected by a line showing mean difference (treatment effect), box limits = 25^th^ and 75^th^ percentiles, center lines = medians, and whiskers = most extreme score within 1.5x the interquartile range.

For both Doppler and NIRS methods (**Figure 5**, **Supplementary Table S3**), evidence favored no group x time interaction (Doppler: BF_10_ = 0.065, 1.5% error, BF_incl_ = 1.070; NIRS: BF_10_ = 0.056, 2.8% error, BF_incl_ = 0.492). Evidence favored no sex-based effect modification for both Doppler (BF_incl_ = 0.401, 2.6% error) and NIRS (BF_incl_ = 0.434, 2.5% error). For both methods, estimated treatment effects are shown in **Table 4** and observed changes are in **Supplementary Table S3**. For peak femoral artery hyperemic velocity, 1 female and 2 male participants in the training group were removed due to poor signal quality on at least one measurement occasion. For the NIRS method, 3 female participants were excluded from analysis for having adipose tissue thickness greater than 2.0 cm (1 allocated to the control group and 2 from the treatment group), and 1 male participant allocated to the treatment group was excluded due to poor signal quality. For included participants, thigh ATT was 1.08 (0.38), Min: 0.38, Max: 1.91 cm.

**Table 4:** Posterior estimates of treatment effects and modification by sex for local/trained limb vascular outcomes.

Outcome	Pre-to-mid (Training Δ -Control Δ)	Pre-to-mid modification by sex (M-F)	Pre-to-post (Training Δ -Control Δ)	Pre-to-post modification by sex (M-F)
Femoral Peak Hyperemic Velocity (cm/s)	6.05 [-3.46 to 15.68]	-9.94 [-29.09 to 8.74]	-4.74 [-14.21 to 4.95]	-12.55 [-31.19 to 6.57]
Thigh NIRS Reperfusion Slope (StO_2_ %/s)	0.04 [-0.18 to 0.25]	0.19 [-0.23 to 0.61]	0.18 [-0.03 to 0.40]	0.32 [-0.11 to 0.73]
PLM Hyperemia (mL/min)	-9.29 [-157.87 to 134.10]	-15.32 [-310.14 to 270.97]	33.02 [-118.12 to 174.70]	36.90 [-251.48 to 331.29]

Treatment effects are estimates of the training group Δ minus the control group Δ between the denoted time points. Modification by sex reflects the difference in the treatment effect between males and females for the denoted time point (i.e., training group Δ – control group Δ for males minus the training group Δ – control group Δ for females). Values are the median of the posterior effect distribution [95% credible interval]. NIRS, near-infrared spectroscopy; StO_2,_ tissue oxygen saturation; PLM, passive leg movement.

### Passive leg movement hyperemia

There was evidence against a group x time interaction (BF_10_ = 0.007, 1.6% error, BF_incl_ = 0.148), and evidence against effect modification by sex (BF_incl_ = 0.206, 3.3% error). Estimated treatment effects are shown in **Table 4** and observed changes are in **Supplementary Table S3**. Data from 3 participants (2 male, 1 female) in the training group were excluded due to poor signal quality/movement artifact during the test on at least one testing visit.

### Cardiorespiratory fitness

All participants met 2 or more criteria for achieving a maximal test in both the pre and post testing visits. Cardiorespiratory fitness was not assessed at the mid-intervention time point. There was evidence against a group x time interaction for VO_2max_ (BF_10_ = 0.048, 2.9% error, BF_incl_ = 0.394), VT (BF_10_ = 0.029, 4.9% error, BF_incl_ = 0.336), and RCP (BF_10_ = 0.018, 1.9% error, BF_incl_ = 0.298), but not time to exhaustion (TTE) (BF_10_ = 0.595, 3.0% error, BF_incl_ = 2.59). The BF_incl_ for TTE indicated the group x time interaction increased model probability beyond main effects of group and time. There was no evidence of effect modification by sex for any cardiorespiratory fitness outcomes (VO_2max_, BF_incl_ = 0.964, 4.8% error, VT, BF_incl_ = 0.501, 4.9% error, RCP, BF_incl_ = 0.409, 4.4% error, TTE, BF_incl_ = 0.361, 5.2% error). Estimated treatment effects are shown in **Table 5**. Two male participants in the training group exhibited irregular breathing responses and were excluded from analysis for both VT and RCP, and 2 additional participants (1 female, 1 male, both in training group) had irregular breathing responses near VT during at least one test and were thus excluded from analysis. Observed changes in cardiorespiratory fitness outcomes were VO_2max_, control Δ: 0.05 (0.14) L/min; training Δ: 0.00 (0.19) L/min, VT, control Δ: 0.02 (0.18) L/min; training Δ: -0.01 (0.16) L/min, RCP, control Δ: -0.01 (0.20) L/min; training Δ: 0.00 (0.24) L/min, and TTE control Δ: -8.3 (27.3) s; training Δ: 9.2 (29.2) s (**Supplementary Table S4**).

**Table 5:** Posterior estimates of treatment effects and modification by sex for cardiorespiratory outcomes.

Outcome	Pre-to-post (Training Δ-Control Δ)	Pre-to-post modification by sex (M-F)
VO_2max_ (L/min)	-0.04 [-0.14 to 0.06]	-0.13 [-0.33 to 0.07]
VT (L/min)	-0.04 [-0.14 to 0.06]	-0.08 [-0.28 to 0.13]
RCP (L/min)	0.01 [-0.12 to 0.14]	-0.05 [-0.30 to 0.20]
TTE (s)	17.5 [1.0 to 33.4]	-3.4 [-35.9 to 30.1]

Treatment effects are estimates of the training group Δ minus the control group Δ between the denoted time points. Modification by sex reflects the difference in the treatment effect between males and females for the denoted time point (i.e., training group Δ – control group Δ for males minus the training group Δ – control group Δ for females). Values are the median of the posterior effect distribution [95% credible interval]. VO_2max_, maximal oxygen uptake; VT, ventilation threshold; RCP, respiratory compensation point; TTE, time to exhaustion.

### Non-invasive muscle oxidative capacity

Thirty-one total participants were retained in the final analysis (n = 17 training, n = 14 control). Three female participants were excluded from analysis for having adipose tissue thickness greater than 2.0 cm, and a further 9 were excluded for poor signal quality and/or variable StO_2_ responses during the brief occlusions in at least one of the three testing occasions. Three males were excluded for the same reasons. There was evidence against a group x time interaction (BF_10_ = 0.018, 1.4% error, BF_incl_ = 0.212), and evidence against effect modification by sex (BF_incl_ = 0.288, 2.3% error). The estimated treatment effect from pre-to-mid was -3.5 sec [-12.3 to 5.0] sec and pre-to-post was -1.9 sec [-10.5 to 6.8] sec. Observed changes are in **Supplementary Table S4**.

## 4 Discussion

The aims of this investigation were to assess the effects of single-sprint training on local and/or systemic vascular function, and to further investigate changes in maximal oxygen uptake and components of oxygen transport in young, sedentary individuals. In partial agreement with our hypotheses and previous work demonstrating that a 4-week training intervention utilizing a single 20-second sprint effort performed 3 days per week did not improve VO_2max_ (Songsorn et al., 2016), no improvements in maximal oxygen uptake were observed in the current study. In contrast to our hypotheses, however, no changes in vascular function, ventilatory thresholds, or thigh muscle oxidative capacity were observed, with evidence collectively being against a training effect. The null findings of this study are strengthened by the effective implementation of the training intervention and rigorous use of several non-invasive measures of vascular and metabolic function that all point to a null effect of the intervention. To advance future investigation into the cardioprotective benefits of brief exercise bouts, it is therefore important to reexamine our hypotheses in light of these results (Miller-Halegoua, 2017; Axford et al., 2022).

The finding that vascular function outcomes were not affected by single-sprint training is in contrast with numerous studies demonstrating improved local and systemic conduit artery endothelial function and microvascular function following other exercise training interventions in young, healthy individuals (Clarkson et al., 1999; Alomari and Welsch, 2007; Rakobowchuk et al., 2008; Tinken et al., 2010; Birk et al., 2012; Chidnok et al., 2020; Williams et al., 2020; Kranen et al., 2023). Importantly, there is a substantial difference in exercise volume between the interventions utilized in these studies and that of the current one. For example, Rakobowchuk et al. (2008) reported improved popliteal FMD and distensibility following 6 weeks of both sprint interval training (i.e., 4–6 x 30-second sprint efforts performed 3 days per week) and continuous moderate intensity training (i.e., 40–60 minutes at 65% VO_2max_ performed 5 days per week). Clarkson et al. (1999) reported improved brachial FMD in male military recruits after a 10-week training program consisting of daily 3-mile runs and resistance exercises. Tinken et al. (2010) and Alomari and Welsch (2007) utilized handgrip exercise interventions (e.g., 20-30-minute bouts performed 4–5 days per week) leading to improved brachial FMD (Tinken et al., 2010) and forearm reactive hyperemic flow (Alomari and Welsch, 2007). Kranen et al. (2023) observed that after 4 weeks of high-intensity interval running training (e.g., 12 x 1-minute efforts performed 3 days per week), brachial artery FMD (but not peak reactive hyperemia) improved. Similarly, Williams et al. (2020) demonstrated reproducible improvement in brachial FMD following two separate 4-week treadmill high-intensity interval training interventions (e.g., each bout lasting 40 minutes). Such differences in training volume likely explain the lack of improvement in the current study.

Given the mechanistic role of exercise-induced hemodynamic stimuli in driving vascular adaptation (Green et al., 2017), it would seem that the shear stimulus evoked by the single-sprint training was of insufficient magnitude or duration. The shear stimulus generated by sprint exercise has not been extensively investigated, although observations from our prior work (Chatlaong et al., 2025) demonstrate marked increases in shear rate occurring non-locally in the brachial artery (peak shear rate values > 175 ^-s^). Importantly, however, this stimulus may not last long enough, with mean shear rate returning toward baseline within a few minutes of passive recovery after a single sprint. Although measures of the shear stimulus in the legs were not made in this study, previous work suggests that a single 30s sprint can acutely elicit altered shear rate post-exercise in the femoral artery (DeBlois et al., 2020), although the magnitude and time course throughout the immediate post-exercise recovery period remain somewhat unclear. To illustrate the necessity of an adequate shear stimulus, Tinken et al. (2010) used a bilateral handgrip training model, where a pneumatic cuff was placed on one forearm (60 mmHg) to attenuate exercise-induced increases in shear rate during training. They observed improved reactive hyperemia and brachial FMD at 2-, 4-, and 6- weeks of training in the free-flow arm, though not in the cuffed arm. Similar results were observed in a subsequent cycle training study (Birk et al., 2012), demonstrating the need for shear stimuli to evoke adaptation. Taken together with the results of the current training study, we would posit that a brief exposure to high shear rate magnitude alone is ineffective for improving vascular function, and that a greater duration of hemodynamic stimulus is needed. To our knowledge this has not been previously reported for an intense effort in isolation. This is of significant interest when considering whether interventions of brief, intense exercise can improve vascular function. Changes should be made to the current intervention when targeting sedentary young, healthy, adults. The addition of either more sprint repetitions, or a period of continuous exercise immediately following the sprint, could help maintain high metabolic rate and cardiac output, translating to more prolonged exposure to high shear rates.

Secondary to vascular outcomes, we hypothesized that substantial local muscle fatigue and metabolic stress induced by even a single maximal sprint effort (Bogdanis et al., 1996) could drive metabolic adaptations within the trained muscles, reflected in greater muscle oxidative capacity and/or metabolic rates at ventilatory thresholds. This hypothesis, however, was also not supported by our findings as these outcomes did not change. Previous studies utilizing muscle biopsy techniques demonstrate upregulation in markers of muscle oxidative capacity in the vastus lateralis following larger volumes of sprint interval training (Burgomaster et al., 2005; Gibala et al., 2006; Burgomaster et al., 2008; Gillen et al., 2014, 2016). Moreover, acutely, PGC-1α expression was shown to increase in the vastus lateralis following a bout of 2 x 20s sprints (Metcalfe et al., 2015), indicating a potential for driving adaptations related to muscle oxidative capacity. Accordingly, these findings indicate that similarly to vascular function, a greater volume of exercise appears necessary to improve muscle oxidative capacity. However, a recent study in healthy young adults also found that non-invasively measured muscle oxidative capacity did not change with larger sprint interval training volumes (i.e., up to 6 x 30-second sprint efforts performed 3 days per week) despite improved VO_2max_ (Inglis et al., 2025) and ventilatory thresholds (Inglis et al., 2024). While the lack of improvement in muscle oxidative capacity in the current study could be attributable to a lack of training volume, considering this evidence leads us to further reexamine our hypothesis that muscle oxidative capacity may readily improve with minimal exercise doses independently of changes in VO_2max_.

The current study is not without limitations. First, our study is limited in generalizability to clinical populations or older adults. There is some evidence that vascular function could improve more easily in populations with poor vascular function at study onset (Ashor et al., 2014; Khalafi et al., 2022). Our target population of young, healthy individuals was based on the need for interventions to improve vascular function early in life (Olsen et al., 2016). Moreover, the inclusion of only one intervention arm performing the single-sprint training as opposed to including another group performing 2 x 20 second protocols limits the ability of the current study to evaluate the dose-response relationship between sprint exercise and cardiometabolic adaptations. However, the findings of the current study provide further rationale for such designs in future research. Secondly, some methodological considerations warrant discussion. We did not standardize or experimentally control for menstrual cycle phase or hormonal birth control use, which may affect vascular reactivity (Adkisson et al., 2010). The results in **Table 2** demonstrate a relatively even between-group distribution of participants beginning the study in each status, which would help prevent systematic bias in training effects or modification by sex (i.e., the focus of the study). Moreover, although average peak FMD response times have been shown to occur between 1–3 minutes post-deflation (Peretz et al., 2007; Black et al., 2008; Thijssen et al., 2008), it is possible that a small number of participants exhibit peak responses in 25–30 seconds (Peretz et al., 2007). While our measures were taken after 30 seconds, we would not expect this to affect study conclusions. Another consideration is that the current study did not assess conduit artery endothelial function in legs (trained limbs). Measures of SFA or popliteal artery FMD, which are commonly used to assess conduit artery endothelial function following exercise training (Rakobowchuk et al., 2008; Hunt et al., 2013; Green et al., 2025), were not included. While limiting, this was due to the use of proximal thigh cuff occlusion (fitted to the uppermost portion of the thigh), required for NIRS-derived PORH outcomes to be measured in the vastus lateralis (i.e., a trained muscle), which may have prevented assessment of either artery in response to distal cuff occlusion as is recommended for FMD. Lastly, it should be acknowledged that our results for muscle oxidative capacity are limited by missing data, which may be due to participant characteristics or protocol differences. Given the aforementioned findings of Inglis et al. (2025), we find it unlikely that our conclusions would differ if all participants were retained in the final analysis.

## Conclusion

This study corroborates previous evidence that exercise training consisting of a single 20-second, maximal cycling sprint with no warmup or cool down does not elicit improvements in maximal oxygen uptake. It further demonstrates that vascular function, ventilatory thresholds, and muscle oxidative capacity also do not improve, nor does the treatment effect depend on sex. Given the growing interest in utilizing brief isolated bouts of intense exercise, and few investigations into their effects on vascular function, this work establishes an important basis that helps advance future research in this area.

## Data Availability

The raw data supporting the conclusions of this article will be made available by the authors, without undue reservation.

## Footnotes

1 BF_incl_ differs from BF_10_ reported for model comparison. BF_10_ compares the probability of the interaction model predicting the data to the probability of the null model predicting the data, after considering all other possible models (null, main effects, etc.). BF_incl_ focuses more directly on the addition of an interaction, and whether this improves or penalizes model probability in predicting the data when compared to a model that already contains main effects of the lower order terms. Accordingly, BF_incl_ might suggest that adding an interaction improves model probability, but the full factorial model may still be unlikely to explain the data when compared to the null or other candidate models.

2 Using this approach as the main form of testing is recommended when there are many “nuisance” candidate models containing lower order terms that are not of substantive theoretical interest (Hinne et al., 2020).
