# Supplementary material for: Cardiorespiratory and vascular function outcomes following 4 weeks of single-sprint training
Source: Front Physiol. 2026 May 29;17:1741749. doi: 10.3389/fphys.2026.1741749 (PMC13259722; doi:10.3389/fphys.2026.1741749)
Supplement: Supplementary file 1 [file Table1.docx]

| **Table S1**: Standardized estimates of treatment effects and modification by sex. | | | | |
| --- | --- | --- | --- | --- |
| **Outcome** | **Pre-to-mid (Training Δ -Control Δ)** | **Pre-to-mid Modification by Sex (M-F)** | **Pre-to-post (Training Δ -Control Δ)** | **Modification by Sex (M-F)** |
| Brachial Peak Hyperemic Velocity (cm/s) | 0.34 [-0.41 to 1.11] | 0.35 [-1.16 to 1.87] | 0.22 [-0.55 to 0.97] | -0.02 [-1.53 to 1.49] |
| Forearm Reperfusion Slope (StO_2_ %/s) | -0.01 [-0.83 to 0.79] | -0.55 [-2.17 to 1.06] | 0.24 [-0.58 to 1.04] | -0.59 [-2.21 to 1.02] |
| Brachial FMD (mm) | 0.24 [-0.56 to 1.02] | -0.93 [-2.49 to 0.64] | -0.37 [-1.17 to 0.42] | 0.72 [-0.85 to 2.27] |
| Systolic Blood Pressure (mmHg) | -0.02 [-0.71 to 0.69] | 0.53 [-0.91 to 1.97] | -0.29 [-1.00 to 0.40] | 1.10 [-0.34 to 2.54] |
| Diastolic Blood Pressure (mmHg) | -0.05 [-0.78 to 0.67] | -0.66 [-2.14 to 0.78] | -0.33 [-1.05 to 0.39] | 0.35 [-1.14 to 1.79] |
| Femoral Peak Hyperemic Velocity (cm/s) | 0.52 [-0.29 to 1.34] | -0.86 [-2.53 to 0.76] | -0.40 [-1.21 to 0.42] | -1.09[-2.71 to 0.57] |
| Thigh Reperfusion Slope (StO_2_ %/s) | 0.13 [-0.64 to 0.89] | 0.70 [-0.86 to 2.27] | 0.66 [-0.11 to 1.42] | 1.18 [-0.41 to 2.73] |
| PLM Hyperemia (mL/min) | -0.05 [-0.85 to 0.73] | -0.08 [-1.70 to 1.48] | 0.18 [-0.64 to 0.94] | 0.20 [-1.38 to 1.81] |
| VO_2max_ (L/min) | - | - | -0.35 [-1.15 to 0.46] | -1.06 [-2.66 to 0.55] |
| VT (L/min) | - | - | -0.30 [-1.13 to 0.53] | -0.65 [-2.30 to 1.04] |
| RCP (L/min) | - | - | 0.07 [-0.76 to 0.87] | -0.33 [-1.97 to 1.33] |
| TTE (s) | - | - | 0.86 [0.05 to 1.64] | -0.17 [-1.73 to 1.46] |
| Muscle Oxidative Capacity Recovery Time Constant (s) | -0.40 [-1.40 to 0.57] | 0.54 [-1.53 to 2.58] | -0.21 [-1.19 to 0.77] | 0.63 [-1.42 to 2.69] |
| Treatment effects are estimates of the training group Δ minus the control group Δ between the denoted time points. Modification by sex reflects the difference in the treatment effect between males and females for the denoted time point (i.e., training group Δ – control group Δ for males minus the training group Δ – control group Δ for females). Values are the median of the posterior effect distribution [95% credible interval]. FMD = flow mediated dilation; StO_2_ = tissue oxygen saturation; PLM = passive leg movement; VO_2max_ = maximal oxygen uptake; VT = ventilation threshold; RCP = respiratory compensation point; TTE = time to exhaustion. | | | | |

| **Table S2**: Observed values by group for systemic/non-local vascular outcomes. | | | | | | |  |
| --- | --- | --- | --- | --- | --- | --- | --- |
| **Outcome** | **Group** | **Pre** | **Mid** | **Pre-to-mid Δ** | **Post** | **Pre-to-post Δ** |  |
| Brachial Hyperemic Velocity (cm/s) | Control (n = 22) | 65.19 (11.43) | 65.12 (12.47) | -0.06 (8.07) | 63.59 (16.14) | -1.59 (10.06) |  |
|  | Training (n = 24) | 61.49 (12.27) | 64.41 (12.81) | 2.92 (13.08) | 61.84 (11.81) | 0.35 (11.61) |  |
| Forearm Reperfusion Slope (StO_2_ %/s) | Control (n = 22) | 1.59 (0.47) | 1.62 (0.52) | 0.03 (0.58) | 1.46 (0.49) | -0.13 (0.49) |  |
|  | Training (n = 22) | 1.43 (0.59) | 1.46 (0.75) | 0.03 (0.60) | 1.44 (0.59) | -0.03 (0.61) |  |
| Resting Brachial Diameter (mm) | Control (n = 22) | 3.637 (0.542) | 3.69 (0.551) | 0.005 (0.021) | 3.631 (0.513) | -0.001 (0.016) |  |
|  | Training (n = 24) | 3.704 (0.613) | 3.704 (0.577) | 0.000 (0.022) | 3.735 (0.641) | 0.003 (0.025) |  |
| Peak Brachial Diameter (mm) | Control (n = 22) | 3.820 (0.529) | 3.838 (0.537) | 0.002 (0.025) | 3.804 (0.534) | -0.002 (0.016) |  |
|  | Training (n = 24) | 3.877 (0.661) | 3.863 (0.615) | -0.001 (0.027) | 3.866 (0.703) | -0.001 (0.024) |  |
| Brachial FMD (mm) | Control (n = 22) | 0.183 (0.101) | 0.148 (0.102) | -0.035 (0.142) | 0.173 (0.073) | -0.010 (0.108) |  |
|  | Training (n = 24) | 0.173 (0.099) | 0.159 (0.100) | -0.014 (0.096) | 0.131 (0.136) | -0.042 (0.121) |  |
| Brachial FMD (%) | Control (n = 22) | 5.2 (3.2) | 4.2 (3.1) | -1.1 (3.7) | 4.8 (1.9) | -0.5 (3.2) |  |
|  | Training (n = 24) | 4.6 (2.7) | 4.3 (2.6) | -0.3 (2.7) | 3.4 (3.2) | -1.2 (3.4) |  |
| Systolic Blood Pressure (mmHg) | Control (n = 22) | 113.1 (16.2) | 112.0 (14.2) | -1.1 (9.7) | 113.5 (13.0) | 0.4 (8.1) |  |
|  | Training (n = 24) | 115.5 (12.7) | 114.2 (13.1) | -1.2 (6.8) | 114.1 (13.3) | -1.4 (7.3) |  |
| Diastolic Blood Pressure (mmHg) | Control (n = 22) | 76.3 (7.0) | 75.0 (7.3) | -1.2 (7.2) | 76.1 (7.3) | -0.2 (5.6) |  |
|  | Training (n = 24) | 77.6 (6.9) | 76.1 (7.8) | -1.5 (6.2) | 75.7 (7.6) | -1.9 (6.3) |  |
| Values at each time point are mean (SD). FMD = flow mediated dilation; StO_2_ = tissue oxygen saturation. | | | | | | | |

| **Table S3**: Observed values by group for local/trained limb vascular outcomes. | | | | | | | |  |
| --- | --- | --- | --- | --- | --- | --- | --- | --- |
| **Outcome** | **Group** | **Pre** | **Mid** | **Pre-to-mid Δ** | **Post** | **Pre-to-post Δ** |  |  |
| Femoral Peak Hyperemic Velocity (cm/s) | Control (n = 22) | 76.69 (16.21) | 71.66 (11.97) | -5.03 (17.40) | 80.2 (16.78) | 3.51 (15.92) |  |  |
|  | Training (n = 21) | 82.97 (18.6) | 84.03 (16.78) | 1.06 (17.88) | 81.77 (17.93) | -1.20 (10.88) |  |  |
| Thigh Reperfusion Slope (StO_2_ %/s) | Control (n = 21) | 1.10 (0.47) | 1.05 (0.57) | -0.04 (0.30) | 1.07 (0.53) | -0.03 (0.29) |  |  |
|  | Training (n = 21) | 1.12 (0.51) | 1.11 (0.45) | -0.01 (0.38) | 1.27 (0.60) | 0.15 (0.38) |  |  |
| Resting Femoral Artery Blood Flow (ml/min) | Control (n = 21) | 184.88 (76.16) | 178.65 (114.78) | -6.23 (78.74) | 174.94 (62.11) | -9.94 (64.52) |  |  |
|  | Training (n = 21) | 225.30 (118.87) | 205.20 (92.14) | -20.09 (130.25) | 173.81 (59.53) | -51.49 (109.45) |  |  |
| Peak PLM Femoral Artery Blood Flow (ml/min) | Control (n = 22) | 581.31 (257.74) | 529.59 (292.64) | -51.73 (267.30) | 538.52 (252.35) | -42.79 (242.77) |  |  |
|  | Training (n = 21) | 634.44 (312.71) | 559.79 (274.58) | -74.65 (346.66) | 583.21 (248.61) | -51.23 (290.19) |  |  |
| PLM Hyperemia (mL/min) | Control (n = 22) | 396.43 (254.48) | 350.94 (271.65) | -45.50 (245.03) | 363.59 (236.95) | -32.84 (232.36) |  |  |
|  | Training (n = 21) | 409.15 (234.05) | 354.59 (231.50) | -54.56 (266.72) | 409.40 (220.65) | 0.26 (241.23) |  |  |
| Values at each time point are mean (SD). StO_2_ = tissue oxygen saturation; PLM = passive leg movement. | | | | | | | | |

| **Table S4**: Observed values by group for cardiorespiratory outcomes. | | | | | | |  |  |
| --- | --- | --- | --- | --- | --- | --- | --- | --- |
| **Outcome** | **Group** | **Pre** | **Mid** | **Pre-to-mid Δ** | **Post** | **Pre-to-post Δ** | |  |
| VO_2max_ (L/min) | Control (n = 22) | 2.12 (0.67) | - | - | 2.17 (0.72) | 0.05 (0.14) |  |  |
|  | Training (n = 24) | 2.10 (0.53) | - | - | 2.10 (0.58) | 0.00 (0.19) |  |  |
| VT (L/min) | Control (n = 22) | 1.23 (0.24) | - | - | 1.26 (0.27) | 0.02 (0.18) |  |  |
|  | Training (n = 20) | 1.22 (0.26) | - | - | 1.21 (0.28) | -0.01 (0.16) |  |  |
| RCP (L/min) | Control (n = 22) | 1.60 (0.48) | - | - | 1.59 (0.51) | -0.01 (0.20) |  |  |
|  | Training (n = 22) | 1.58 (0.40) | - | - | 1.57 (0.39) | 0.00 (0.24) |  |  |
| TTE (s) | Control (n = 22) | 437.9 (101.0) | - | - | 429.6 (105.7) | -8.3 (27.3) |  |  |
|  | Training (n = 24) | 436.6 (91.2) | - | - | 445.9 (100.4) | 9.2 (29.2) |  |  |
| Muscle Oxidative Capacity Recovery Time Constant (s) | Control (n = 14) | 26.9 (13.2) | 26.9 (11.7) | -0.0 (9.7) | 24.8 (9.7) | -2.1 (13.8) |  |  |
|  | Training (n = 17) | 27.0 (13.6) | 23.4 (8.0) | -3.6 (16.4) | 23.0 (6.7) | -4.0 (13.7) |  |  |
| Values at each time point are mean (SD). VO_2max_ = maximal oxygen uptake; VT = ventilation threshold; RCP = respiratory compensation point; TTE = time to exhaustion. | | | | | | | | |
